# Complete genome sequence of carbapenem-resistant pathogenic *Klebsiella aerogenes* strain CH7 isolated from vermicompost

**DOI:** 10.1128/mra.01284-23

**Published:** 2024-05-03

**Authors:** Niteesh Kumar Pandey, Saugata Hazra

**Affiliations:** 1 Department of Bioscience and Bioengineering, Indian Institute of Technology-Roorkee, Roorkee, Uttarakhand, India; 2 Centre for Nanotechnology, Indian Institute of Technology-Roorkee, Roorkee, Uttarakhand, India; California State University San Marcos, San Marcos, California, USA

**Keywords:** *Klebsiella*, carbapenem resistance, metalloenzymes

## Abstract

We announce the complete genome of *Klebsiella aerogenes* strain CH7, isolated from a vermicompost sample. A total of 9.14131 million high-quality reads comprised 96 contigs with 5,273 genes and 5,038 protein-coding genes.

## ANNOUNCEMENT

*Klebsiella aerogenes* strain CH7 was isolated from ready-to-use fully matured vermicompost, collected in March 2021 from Janjgir (22.0090841″N 82.578896″E), Chhattisgarh, India. To estimate the total bacterial load, the sample was serially diluted and plated on nutrient agar (HiMedia). HiCrome Klebsiella Selective Agar Base (HiMedia) was used to isolate *Klebsiella aerogenes*. Ampicillin (15 µg/mL), kanamycin (30 µg/mL), and trimethoprim (6 µg/mL) antibiotics-treated Muller-Hinton agar (HiMedia) plates were used to screen drug-resistant isolate (1, 2). All agar-based bacterial growth and isolation were performed at 37°C for 24 h (1, 3). *K. aerogenes* strain CH7 isolate was sensitized by the disk diffusion method. It showed resistance against cefpodoxime (10 µg), vancomycin (30 µg), rifampicin (5 µg), clindamycin (2 µg), and augmentin (30 µg) and last-resort carbapenems: imipenem (10 µg), and meropenem (10 µg) disk (4–6). It was confirmed as an extended spectrum β-lactamase (ESBL) producer by using the cefepime plus clavulanate combination disk test (7).

Isolate was grown in LB Broth at 37°C for 24 h (HiMedia), and genomic DNA was isolated by using QIAcube HT Kit (Qiagen). The TruSeq Nano DNA Library Kit (Illumina) was used for WGS library preparation. Illumina Hi-Seq 2000 platform was used for sequencing and provided 10.04416 million raw reads (forward + reverse), with a read length of 151 bp. By using fastp 0.20.0 tool (8), quality filtering was performed, and a total of 9.14131 million high-quality (HQ) reads (forward and reverse) with 137 bp HQ read length were obtained. SPAdes 1.0.4 (9) sequence assembler was used for primary assembly (Table 1) and provided contigs with minimum and maximum sequence lengths of 206 and 528,380 bp. The location of 1,536 base pairs 16s rRNA gene in the genome was predicted by barrnap 0.9 tool (10). NCBI-BLAST (blastn) search identified 99.80% identity with *Klebsiella aerogenes* as the closest genome. An assembled genome fasta file was used to create a draft genome assembly, and filtered HQ reads were aligned to the respective assembled genome using aligner Bowtie2 (v 2.3.4.1) (11). Gene annotation was performed by NCBI Prokaryotic Genome Annotation Pipeline version 6.6 (Table 1) (12). The presence of fimbriae type 1, MDR (OqxB and EmrD), and siderophores (salmochelin and enterobactin) genes defines the virulence of the isolate (13). The detection of ampC (CMY2-MIR-ACT-EC Family) and metallo-beta-lactamase genes defines the ESBL and carbapenem resistance phenotype of the strain. All bioinformatics tools were used in default parameters unless there were specific requirements.

**TABLE 1:** Summary of final draft assembly and annotation statistics

Metrics	Statistics
Total sequence length (bp)	5,385,268
Number of contigs	96
*N*_50_ contig (kb)	220.3
*L*_50_ contig	8
GC (%)	54.5
Genes	5,273
Protein-coding genes	5,038

## Data Availability

The genome sequence draft of *Klebsiella aerogenes* strain CH7 can be found in the National Centre for Biotechnology Information (NCBI). Whole Genome Sequence data have been deposited at GenBank under accession JAXIVP000000000. The version described in this paper is JAXIVP010000000. BioProject accession number is PRJNA1023395 and SRA number is SRR26362847. 16s rRNA sequence used for nearest reference check could be found under GenBank accession number OR619830.
